# Promoter hypermethylation and silencing of tissue factor pathway inhibitor-2 in oral squamous cell carcinoma

**DOI:** 10.1186/s12967-014-0237-7

**Published:** 2014-09-02

**Authors:** Yi-Hui Lai, Ru-Yin He, Jian-Liang Chou, Michael W-Y Chan, Yu-Fen Li, Chien-Kuo Tai

**Affiliations:** Department of Life Science and Institutes of Molecular Biology and Biomedical Science, National Chung Cheng University, Min-Hsiung, Chia-Yi, Taiwan; Institute of Biostatistics, China Medical University, Taichung, Taiwan

**Keywords:** Oral squamous cell carcinoma, DNA methylation, Biomarker, TFPI-2, Tumor suppressor gene, Metastasis, Matrix-associated serine protease inhibitor, Matrix metalloproteinase-2

## Abstract

**Background:**

The treatment of oral squamous cell carcinoma (OSCC) following early detection is associated with good outcomes. Therefore, the survival and prognosis of OSCC patients could be hugely improved by identifying reliable biomarkers for the early diagnosis of the disease. Our previous methylation microarray analysis results have suggested that the gene encoding tissue factor pathway inhibitor-2 (TFPI-2) is a potential clinical predictor as well as a key regulator involved in OSCC malignancy.

**Methods:**

Methylation of the *TFPI-2* promoter in oral tissue specimens was evaluated by bisulfite sequencing assay, quantitative methylation-specific PCR, and pyrosequencing assay. The differences in methylation levels among the groups were compared using the Mann–Whitney U test. The area under the receiver operating characteristic curve (AUROC) was used to evaluate the discrimination ability for detecting OSCC. Cellular *TFPI-2* expression was analyzed by quantitative reverse-transcription PCR before and after treatment with 5′-aza-2′-deoxycytidine and trichostatin A, to confirm whether *TFPI-2* was epigenetically silenced in OSCC cells. We investigated whether TFPI-2 plays a role as a tumor suppressor by establishing *TFPI-2*-overexpressing OSCC cells and subjecting them to *in vitro* cellular proliferation, migration, and invasion assays, as well as an *in vivo* metastasis assay.

**Results:**

*TFPI-2* was hypermethylated in OSCC tissues versus normal oral tissues (*P* < 0.0001), with AUROC = 0.91, when using a pyrosequencing assay to quantify the methylation level. *TFPI-2* silencing in OSCC was regulated by both DNA methylation and chromatin histone modification. Restoration of *TFPI-2* counteracted the invasiveness of OSCC by inhibiting the enzymatic activity of matrix metalloproteinase-2, and consequently interfered with OSCC metastasis *in vivo*.

**Conclusions:**

Our data suggest strongly that *TFPI-2* is a down-regulated tumor suppressor gene in OSCC, probably involving epigenetic silencing mechanisms. The loss of *TFPI-2* expression is a key event for oral tumorigenesis, especially in the process of tumor metastasis.

## Background

Oral squamous cell carcinoma (OSCC), which constitutes more than 90% of oral cancers arising from the oral cavity [1], is not only one of the most frequently occurring cancers worldwide, but is also particularly prevalent in Taiwan. Oral cancer has been the fourth leading cause of death from cancer among males in Taiwan since 2003 [2]. As a molecularly heterogeneous disease, OSCC is strongly associated with risk factors including alcohol, tobacco, and betel nut consumption [3–6]. OSCC is a particularly troublesome cancer due to its rapid progression and frequent metastasis [7], causing deaths at an increasing rate in Taiwan [2]. The treatment of oral cancer following early detection is associated with good outcomes, but the 5-year survival rate is <30% among patients with stage IV disease [8]. Therefore, identifying reliable biomarkers for the early diagnosis of OSCC may help to improve the patients’ survival and prognosis.

In addition to genetic alterations such as gene mutation [9], loss of heterozygosity [10], and microsatellite instability [11], epigenetic alterations (particularly hypermethylation in the promoter region of regulatory genes) have been increasingly recognized as key events in oral tumorigenesis [12]. By taking advantage of the genome-wide screening approach, we have recently employed the Illumina GoldenGate Methylation Array to identify methylated genes in OSCC tissues (unpublished data). One of the genes identified with this approach was that encoding tissue factor pathway inhibitor-2 (TFPI-2). With a molecular weight of 27, 31, or 33 kDa, depending upon the level of glycosylation [13], TFPI-2 is synthesized and secreted extracellularly mainly by keratinocytes, fibroblasts, smooth-muscle cells, synoviocytes, and endothelial cells [14–17]. As a known Kunitz-type serine protease inhibitor and placental protein 5 [13,18,19], TFPI-2 counteracts the activity of several extracellular matrix (ECM)-associated serine proteases, including trypsin, plasmin, chymotrypsin, cathepsin G, plasma kallikrein, and the factor VIIa-tissue factor complex [20,21]. Previous studies have demonstrated that TFPI-2 suppresses tumor invasion and metastasis via its inhibitory activity on ECM degradation and remodeling [22,23]. Roles of TFPI-2 in induction of the apoptosis pathway and in angiogenesis have also been demonstrated [24–27]. It has been shown that *TFPI-2* is down-regulated via epigenetic silencing mechanisms including promoter hypermethylation and histone deacetylation in several types of tumor, such as pancreatic ductal adenocarcinoma [28], melanoma [29], hepatocellular carcinoma [30], gastric carcinoma [31], and glioma [32]. The level of *TFPI-2* methylation was also found to differ between preoperative and postoperative saliva DNA in oral cancer patients, highlighting its potential diagnostic value as a biomarker for oral cancer [33].

In the present study we first examined the methylation level of *TFPI-2* in clinical OSCC specimens. Current techniques used to measure DNA methylation, including bisulfite sequencing assay, quantitative methylation-specific PCR (qMSP), and pyrosequencing assay, were applied to uncover the DNA methylation status of the *TFPI-2* promoter region. The methylation level of *TFPI-2* was further statistically analyzed to determine whether there was any correlation with the pathological stages of OSCC patients. We then restored the gene expression of *TFPI-2* in OSCC cell lines by using epigenetic drugs and employing lentivirus vector-mediated gene transfer of *TFPI-2*. Restoration of *TFPI-2* significantly suppressed the invasion and metastasis of OSCC cells. Our data strongly suggest that epigenetic silencing of *TFPI-2* plays an important role in oral tumorigenesis.

## Methods

### Collection of oral tissue specimens and bisulfite conversion of genomic DNA

Normal oral tissues, OSCC tissues and their corresponding non-tumor tissues were obtained from the tissue banks of China Medical University Hospital and Buddhist Tzu Chi General Hospital in Taiwan. Genomic DNA of the tissues was isolated using Gentra Puregene Tissue Kit (Qiagen, Valencia, CA) and 500 ng of genomic DNA was subjected to bisulfite conversion using EZ DNA methylation kit (Zymo Research, Orange, CA). Bisulfite conversed Universal Methylated Genomic DNA (Millipore, Billerica, MA,) was used as in-vitro methylated DNA (IVD) control for the methylation level determined by qMSP and pyrosequencing assays.

### Bisulfite sequencing assay and real-time quantitative methylation-specific PCR

For bisulfite sequencing, the primers targeting the promoter region near the *TFPI-2* transcription start site were used for PCR as previously described [30]. The PCR products were separated by gel electrophoresis, purified with a QIAquick gel extraction kit (Qiagen, Valencia, CA, USA), cloned into the yT&A cloning vector (Yeastern Biotech, Taipei, Taiwan), and sequenced. For real-time qMSP, primers targeting the promoter region of *TFPI-2* were as follows: forward, 5′-ATAAAGCGGGTATTCGGGTC-3′; reverse, 5′-CTCCGCCGATTAAAAAAA-3′. Real-time qMSP was performed using ABI StepOne real-time PCR system according to the manufacturer’s instructions (Applied Biosystems, Forster City, CA). As an input control for real-time qMSP, a DNA fragment devoid of any CpG dinucleotide in *ACTB* was amplified using the following primers: forward, 5′-TGGTGATGGAGGTTTAGTAAGT-3′; reverse, 5′-AACCAATAAAACCTACTCCCTTAA-3′. The extents of methylated *TFPI-2* and *ACTB* were determined by the threshold cycle number for each sample. The percentage of *TFPI-2* methylation was calculated as the ratio of TFPI-2 to ACTB of a sample divided by the same ratio of IVD.

### Pyrosequencing methylation assay

To further verify the results of bisulfite sequencing and qMSP, pyrosequencing primers were designed for the region of interest using Pyromark Assay Design v2.0 (Qiagen): forward, 5′-Bio^+^GGGTGATAGTTTTAGTGTATGAATTAGTT-3′; reverse, 5′-CTAAACAACATCCCCCAATACAACCTC-3′; reverse sequencing primer, 5′-ACTTTCTACTCCAAAC-3′. Pyrosequencing assay was carried out using the PyroMark Q24 System (Qiagen) according to the manufacturer’s instructions.

### Epigenetic drug treatment

1 × 10^6^ OSCC cells were seeded onto 10-cm culture dishes and treated with 0.5 μM or 5 μM 5′-aza-2′-deoxycytidine (5-azaDC) (Sigma, St Louis, MO) for 72 h followed by 0.25 μM trichostatin A (TSA) (Sigma) or DMSO for 12 h. For TSA treatment alone, cells were incubated with DMSO for 72 h followed by 0.25 μM TSA for 12 h. Drugs and culture medium were refreshed every 24 h during the treatments.

### Quantitative reverse-transcription PCR

Total RNA was extracted from cell lines using REzol (Protech, Taipei, Taiwan) according to the manufacturer’s protocol. Complementary DNA (cDNA) was synthesized from 1 μg of total RNA using Superscript III reverse transcriptase (Invitrogen). Quantitative reverse-transcription PCR (qRT-PCR) was performed using ABI StepOne real-time PCR system as the following steps: 95°C for 10 min, followed by 50 cycles of successive incubation at 95°C for 15 sec and at 68°C for 45 sec. *TFPI-2* and *GAPDH* cDNA were amplified with the following primers: *TFPI-2* forward, 5′-GCGATGCTGCTCAGGAG-3′ and reverse, 5′-TCTGCGTGTACCTGTCGTAGTAG-3′; *GAPDH* forward, 5′-TTGACGGTGCCATGGAATTT-3′ and reverse, 5′-GCCATCAATGACCCCTTCATT-3′. *TFPI-2* expression was normalized against that of *GAPDH.*

### Quantitative chromatin immunoprecipitation-PCR

Chromatin immunoprecipitation (ChIP)-PCR was performed as previously described [34]. In brief, 2 × 10^6^ OSCC cells were cross-linked with 1% formaldehyde and washed with PBS in the presence of protease inhibitors. Cells were homogenized and their chromatin was subjected to ChIP using magnetic Dynal beads (Invitrogen) and antibody against acetylated or trimethylated lysine 9 of histone H3 (H3K9) (Millipore, Temecula, CA). Fold-enrichment of amplified DNAs by ChIP was assessed using protocols as previously described [35]. Specific primers targeting the promoter region of *TFPI-2* were as follows: R1-forward, 5′-GCAGGTCATTTCCGTCTAGCTT-3′ and R1-reverse, 5′-ACCTGCCTCCCAAACTTTCTC-3′; R2-forward, 5′-ACCACTTTCCCTCTCTTTTGCT-3′ and R2-reverse, 5′-TCGTAGTAGTAACGGAGAAGTAGGGC-3′.

### Cell lines

The OSCC cell lines OC2 and OCSL [36], derived from Taiwanese male patients who had habits of alcohol drinking, cigarette smoking, and betel nut chewing, were maintained in RPMI medium supplemented with 10% FBS (Invitrogen, Federick, MD). HEK293T cells were grown in DMEM medium supplemented with 10% FBS.

### Plasmid construction and cell infection

The full-length human *TFPI-2* cDNA (~0.7 kb) was cloned from the immortalized ovarian surface epithelia cell line IOSE [37] by RT-PCR with primers 5′-TTTCTCGGACGCCTTGCC-3′ and 5′-GAATGTTTAAAATTGCTTC-3′. The cDNA was introduced into a lentiviral vector plasmid pSin-IRES-GFP (pIG) [38] at the EcoRI and XbaI sites to generate pSin-TFPI2-IRES-GFP (pTIG). The plasmids pIG, pTIG and pSin-FLuc-IRES-GFP (pFIG, a firefly luciferase-expressing lentiviral vector plasmid) were transiently transfected into HEK293T cells and at 48 h posttransfection the lentivirus-containing supernatants were collected for infection. Successful infection was monitored by GFP expression and the infected cells were sorted by FACSAria III Cell Sorter (BD Bioscience, Franklin Lakes, NJ).

### Extracellular matrix protein extraction and immunoblot assay

Cells were grown to ~90% confluence in culture dishes and extracellular matrix (ECM) proteins was harvested from the cells as described by Ehrlich *et al.* [39]. Briefly, cells were washed three times with PBS and lysed through the incubation with PBS containing 0.5% Triton X-100 for 20 min at room temperature. The cells were washed three times with PBS and another three times with 20 nM Tris–HCl [pH 7.4] containing 100 mM NaCl and 0.1% Tween 20. Finally, 200 μl of 1 × SDS-PAGE sample buffer was added to the culture dishes and agitated for 20 min at room temperature. The collected ECM proteins were boiled and 50 μl of aliquots of the extracts was assayed using 12% polyacrylamide gels. The expression of TFPI-2 protein was detected by a polyclonal antibody against TFPI-2 (Santa Cruz Biotechnology, Santa Cruz, CA).

### Cell proliferation assay

The lentiviral vector-infected cells were seeded onto replicate 96-well plates (500 cells per well). Cell proliferation was determined daily with MTS assay using the CellTiter Aqueous One Solution Cell Proliferation Assay kit (Promega, Madison, WI, USA). Relative cell number was measured on an ELISA plate reader with an absorbance set at a wavelength of 490 nm.

### Cell cycle analysis

The lentiviral vector-infected cells were seeded onto replicate 6-well plates (1 × 10^5^ cells per well). After 48 h, the cells were collected, washed, and fixed in 75% ethanol at −20°C for 48 h. The cells were then treated with 0.1 mg/ml RNase A (Macherey-Nagel, Düren, Germany), stained with 10 μg/ml propidium iodide (Sigma), and analyzed by FACSCalibur (BD Biosciences). The percentage of apoptotic cells in sub-G1 area was quantified using CellQuest Pro software (BD Biosciences).

### Matrigel invasion assay

Invasion assay was done on 6-well Transwells (Millipore) with Matrigel-coated polycarbonate filters (8 μm pore size). Two × 10^4^ cells were suspended in 200 μl of RPMI medium supplemented with 0.5% FBS and seeded on the upper chamber well. The lower chamber well was filled with RPMI medium containing 10% FBS. After 48 h of incubation at 37°C, nonpenetrating cells were removed from the upper surface of the filter and penetrating cells on the lower surface of the filter were fixed with methanol and stained with Giemsa solution (Sigma). The numbers of penetrating cells were counted under a light microscope.

### Collagen zymography

The inhibitory effect of TFPI-2 on enzymatic activity of matrix metalloproteinases (MMPs) was assayed by collagen zymography. Two × 10^5^ OC2 cells were suspended in complete medium and plated onto culture dishes. After 48 h of incubation, the medium was changed to serum-free RPMI and the cells were incubated for another 24 h. The conditioned medium was collected and the protein concentrations were determined by BCA Protein Assays (Bio-rad, Hercules, CA). Fifty μg of total proteins was mixed with non-reducing sample buffer containing 315 mM Tris [pH 6.8], 50% glycerol, 5% SDS and 0.025% bromophenol for electrophoresis. Without boiling, the mixed solution was loaded on a 10% polyacrylamide gel containing 0.5 mg/ml collagen (Sigma). After electrophoresis, gel was incubated for 1 h at 25°C in a 2.5% Triton X-100 solution followed by 20-min wash with deionized water for 2 times, and then incubated overnight at 37°C in a 50 mM Tris–HCl [pH 8.0], containing 5 mM CaCl_2_. The gel was stained with 0.25% Coomassie blue and destained with 10% methanol and 7% acetic acid. Enzymatic activity attributed to MMPs can be visualized as clear bands against a blue background. The relative intensity of the bands was quantified by ImageJ.

### *In vivo* tumor metastasis assay

Athymic BALB/c nude mice were obtained from National Laboratory Animal Center, Taiwan. Cells at a density of 1 × 10^6^ in 100 μl of PBS were intravenously injected into the tail veins of athymic BALB/c nude mice. Forty days after tumor cell inoculation, the mice were sacrificed and their lungs were excised. The lung tissues were fixed with 10% formaldehyde and the number of pulmonary tumor nodules on each lung was counted.

### Statistical analysis

The Mann–Whitney U test was used to compare the methylation levels between or among groups of tissue specimen. Receiver operating characteristic (ROC) curve and the area under the ROC curve (AUROC) was calculated to summarize the accuracy of using methylation level for detecting OSCC. The odds ratios between methylation level and OSCC were measured by logistic regression models. Differences with *P* < 0.05 were deemed significant. Analyses were performed in SAS 9.3 (SAS Institute, Cary, NC). Box plots were generated by SigmaPlot version 10 (Systat Software, San Jose, CA).

## Results

### *TFPI-2* is frequently hypermethylated in OSCC tissues

**Figure 1:**
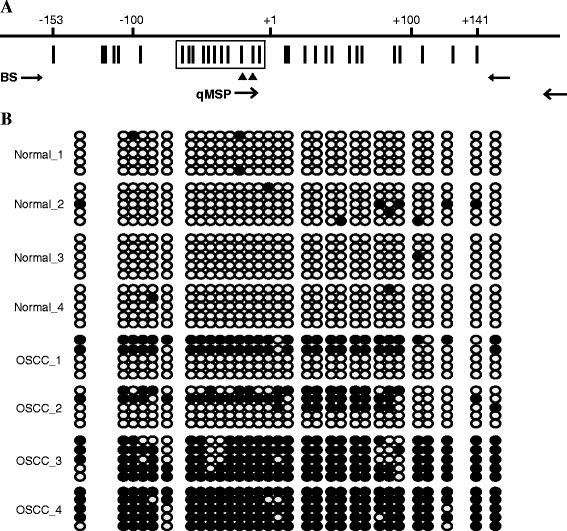
**Bisulfite sequencing of** ***TFPI-2*** **in normal oral and OSCC tissues. (A)** The region centering on the TFPI-2 transcription start site, comprising 31 CpG sites. The distribution of CpG sites (vertical bar) and the position of the hybridized PCR primer pairs for bisulfite sequencing (BS) and quantitative methylation-specific PCR (qMSP) are shown. Two CpG sites in the qMSP forward primer are indicated (filled triangles), and the 11 CpG sites analyzed by pyrosequencing assay are boxed. **(B)** Representative bisulfite sequencing assay for normal oral and OSCC tissues. For each tissue sample, five randomly chosen clones were sequenced and the methylation status of each of the 31 CpG sites is indicated by the circles: closed and open circles represent methylated and unmethylated CpG sites, respectively.

Based on our recent methylation array analysis involving specimens of normal oral tissues and OSCC tissues at different pathological stages, the methylation level of *TFPI-2* was found to differ between normal- and tumor-tissue DNA. The average β values in the three query sites (P152, P9, and E141) were all significantly higher in the tumor tissues than in normal tissues (data not shown). To validate the methylation array data, we further analyzed the methylation status of several oral tissue specimens by bisulfite sequencing assay. As shown in the target region centering on the *TFPI-2* transcription start site comprising 31 CpG sites (Figure 1A), highly dense methylation was observed in the tumor tissues, whereas the normal tissues were essentially free of methylation (Figure 1B).

**Figure 2:**
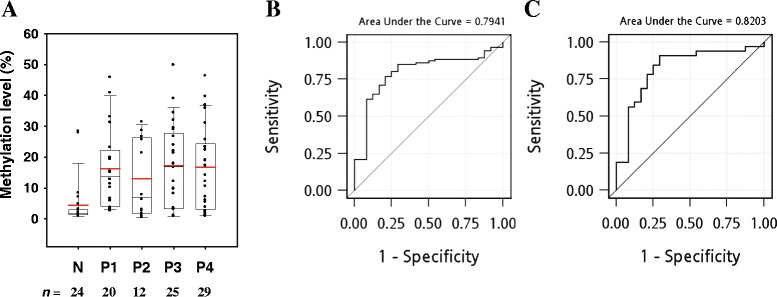
**Methylation status of** ***TFPI-2*** **determined by qMSP assay. (A)** Box plots of the *TFPI-2* methylation level as determined by the qMSP assay. The amount of methylated *TFPI-2* was normalized to the amount of *ACTB* and is expressed as a percentage of that in IVD (given as 100%). N, normal oral tissues; P1–P4, OSCC tissues classified according to pathological stage. In each box plot, the whiskers represent the 10th and 90th percentiles, the lower and upper limits of the box indicate the 25th and 75th percentiles, and the red line is the mean. **(B)** ROC curve obtained using *TFPI-2* methylation to detect OSCC. The AUROC was 0.79. **(C)** ROC curve obtained using *TFPI-2* methylation to detect early-stage OSCC (P1 and P2). The AUROC was 0.82.

**Figure 3:**
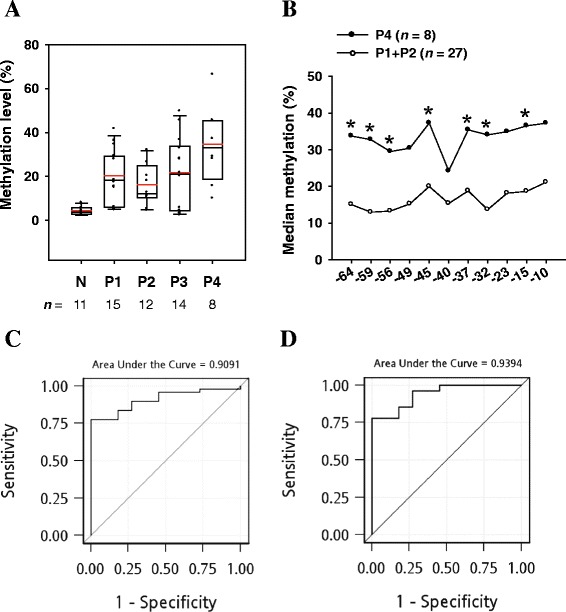
**Methylation status of** ***TFPI-2*** **determined by pyrosequencing assay. (A)** Box plots of *TFPI-2* methylation level determined by the pyrosequencing assay. *TFPI-2* methylation level is expressed as a percentage of that in IVD (given as 100%). The median methylation percentage across all 11 CpG sites was 3.82% in normal oral tissues, and 18.26%, 12.17%, 21.03%, and 33.10% in P1–P4 tumors, respectively. In each box plot, the whiskers represent the 10th and 90th percentiles, the lower and upper limits of the box indicate the 25th and 75th percentiles, and the red line is the mean. **(B)** Differential pattern of *TFPI-2* methylation at each individual CpG site between early-stage (P1 and P2) and late-stage (P4) tissues. Numbers at the *x*-axis indicate nucleotide positions of CpG site relative to the transcription start site. Statistically significant differences in methylation levels are indicated by asterisks (*). **(C)** ROC curve obtained using *TFPI-2* methylation to detect OSCC. The AUROC was 0.91. **(D)** ROC curve obtained using *TFPI-2* methylation to detect early-stage OSCC (P1 and P2). The AUROC was 0.94.

To further quantify the methylation level of *TFPI-2*, we performed real-time qMSP and pyrosequencing methylation assays for oral tissue specimens. The selected patients’ characteristics are presented in Table 1. In total, 110 samples were analyzed by qMSP, while 60 samples were subjected to pyrosequencing methylation assay. The methylation level is summarized as mean and standard deviation values or median and interquartile range values. The 110 oral tissue specimens analyzed by qMSP comprised 86 tumor tissues and 24 normal tissues; the *TFPI-2* methylation level was significantly higher in tumors than in normal tissue (*P* < 0.0001; Figure 2A). The median methylation percentages in tumor tissues at stages P1–P4 were 13.87%, 6.96%, 16.86%, and 16.64%, respectively; the corresponding value in normal tissues was 1.79% (*P* < 0.05 for any specific stage vs the normal tissues). A similar pattern of *TFPI-2* methylation status was obtained using pyrosequencing assay with 60 oral tissue specimens comprising 49 tumor tissues and 11 normal tissues (*P* < 0.0001; Figure 3A). The median methylation percentages in P1–P4 tumors were 18.26%, 12.17%, 21.03%, and 33.10%, respectively, while in normal tissues it was 3.82% (*P* < 0.05 for any specific stage vs the normal tissues). The methylation level differed not only between the normal and tumor tissues, but also between the early- and late-stage tumor tissues. Figure 3B shows the differential pattern of *TFPI-2* methylation levels among the 11 CpG sites between the early-stage (P1 and P2) and late-stage (P4) tumor tissues. The methylation levels were significantly higher in the P4 tumors compared to the P1 and P2 tumors at most of the CpG sites.

**Table 1:** **Selected patients’ characteristics**

**(a) TFPI-2 methylation level by qMSP**						
	**n**	**(%)**	**Mean**	**SD**	**Median**	**IQR**
Gender						
Male	110	(100)	13.64	13.05	8.94	21.06
Pathological Stage						
Normal	24	(21.8)	4.45	7.51	1.79	1.62
I	20	(18.2)	16.23	12.97	13.87	16.71
II	12	(10.9)	12.96	12.46	6.96	23.95
III	25	(22.7)	17.19	13.62	16.86	23.15
IV	29	(26.4)	16.67	13.60	16.64	19.61
Cancer Site						
Buccal	36	(41.9)	17.05	12.46	15.95	18.90
Tongue	50	(58.1)	15.59	13.71	11.66	22.63
**(b) TFPI-2 methylation level by pyrosequencing**						
	**n**	**(%)**	**Mean**	**SD**	**Median**	**IQR**
Gender						
Male	60	(100)	18.80	14.99	15.58	23.79
Pathological Stage						
Normal	11	(18.3)	4.43	1.91	3.82	2.94
I	15	(25.0)	20.35	12.19	18.26	23.14
II	12	(20.0)	16.21	9.48	12.17	12.97
III	14	(23.3)	21.63	16.08	21.03	28.34
IV	8	(13.3)	34.63	18.06	33.10	23.50
Cancer Site						
Buccal	19	(38.8)	29.70	14.41	28.12	16.07
Tongue	30	(61.2)	17.18	12.96	13.86	20.82

SD: standard deviation; IQR: interquartile range.

### *TFPI-2* methylation is a good biomarker for OSCC detection

*TFPI-2* hypermethylation could be used to discriminate tumor tissues from the normal ones. The AUROC values for using methylation level to detect OSCC were 0.79 (95% confidence interval (CI) = 0.69–0.89; Figure 2B) and 0.91 (95% CI = 0.83–0.98; Figure 3C) for the qMSP and pyrosequencing assays, respectively. A 1% increase in methylation level, as quantified by pyrosequencing assay, was associated with a 1.49-fold higher of risk of OSCC (95% CI = 1.06–2.10, *P* = 0.023), while the odds ratio was 1.13 (95% CI = 1.05–1.22, *P* = 0.001) per 1% increase of methylation level when quantified by qMSP. Moreover, *TFPI-2* hypermethylation could be used to discriminate the early-stage tumor tissues from the normal ones. The AUROC values for using methylation level to detect early-stage OSCC (P1 and P2) were 0.82 (95% CI = 0.70–0.94; Figure 2C) and 0.94 (95% CI = 0.87–1.00; Figure 3D) for the qMSP and pyrosequencing assays, respectively.

### Promoter hypermethylation and histone modification contribute to *TFPI-2* inactivation in OCSL cells

**Figure 4:**
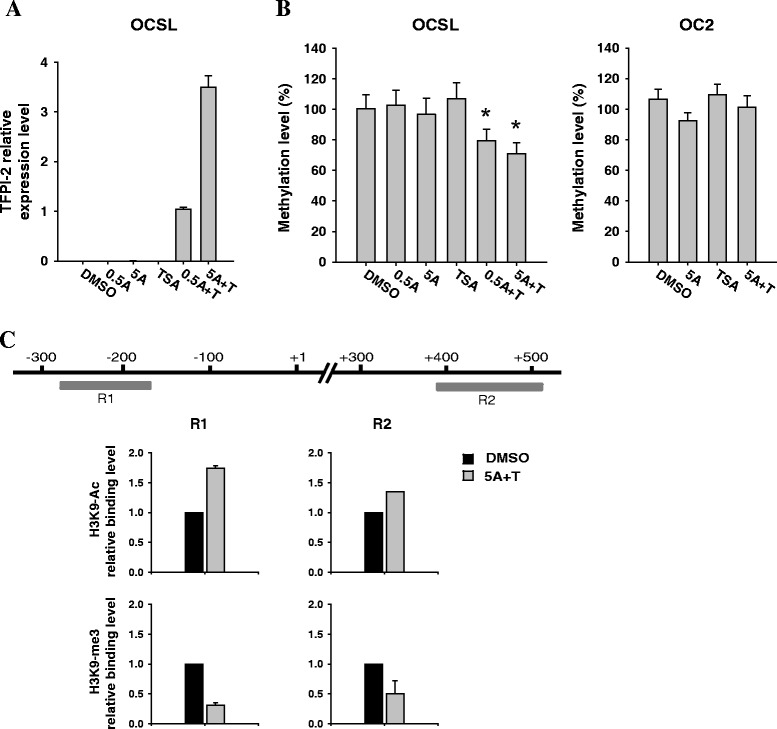
**Reactivation of** ***TFPI-2*** **using epigenetic drugs. (A)** RT-PCR analysis of TFPI-2 expression in OCSL cells treated with 5-azaDC, TSA, or both. 0.5A, 0.5 μM 5-azaDC; 5A, 5 μM 5-azaDC; TSA, 0.25 μM TSA; 0.5A + T, 0.5 μM 5-azaDC and 0.25 μM TSA; 5A + T, 5 μM 5-azaDC and 0.25 μM TSA. The mRNA level of *TFPI-2* in OCSL was compared with that of the control cell line IOSE, which was assigned a value of 1. Error bars indicate standard deviations. **(B)** Methylation levels of OCSL and OC2 cells treated with 5-azaDC, TSA, or both. The DNA of OCSL and OC2 was bisulfite converted and the percentage of *TFPI-2* methylation was determined using the pyrosequencing assay. The *TFPI-2* methylation level is expressed as a percentage of that in IVD (given as 100%). The DMSO- and drug-treated groups were compared using Student’s *t*-test; *P* < 0.05 (*) was considered statistically significant. **(C)** Histone modifications at the promoter region of *TFPI-2* in OCSL cells. ChIP assays were performed with antibodies against H3K9-Ac and H3K9-me3 in OCSL cells. Regions of the *TFPI-2* promoter for quantitative ChIP-PCR assay are indicated as R1 and R2. The binding of H3K9-Ac and H3K9-me3 antibodies to regions R1 and R2 was measured by the quantification of ChIP DNA against a standard curve generated from input DNA. The binding level of each antibody in OCSL treated with 5 μM 5-azaDC and 0.25 μM TSA was compared with that of the cell treated with DMSO, which was assigned a value of 1.

Aberrant DNA methylation in the promoter region is a key mechanism for gene silencing, and so we treated the OSCC cell lines OCSL and OC2 with the demethylating agent 5-azaDC to determine whether *TFPI-2* expression could be restored. We also investigated whether histone modification is involved in *TFPI-2* silencing using the histone deacetylase inhibitor TSA. RT-PCR revealed no detectable *TFPI-2* mRNA expression in OC2 cells irrespective of the treatment (data not shown). In OCSL cells, neither 5-azaDC nor TSA treatment alone induced the expression of *TFPI-2*; however, a dramatic increase in *TFPI-2* expression was observed after combined treatment with 5-azaDC and TSA (Figure 4A). In agreement with *TFPI-2* reactivation in OCSL cells, *TFPI-2* methylation levels in OCSL were decreased when treated with the combination of 5-azaDC and TSA (Figure 4B, left); in contrast, there was no such effect in OC2 cells (Figure 4B, right). To further confirm the involvement of histone modification in *TFPI-2* regulation in OCSL cells, we performed ChIP-PCR assay to analyze histone status in respect to active and repressive chromatin marks at the promoter region of *TFPI-2* by using antibodies against acetylated H3K9 (H3K9-Ac) and trimethylated H3K9 (H3K9-me3). After combined treatment with 5-azaDC and TSA, higher levels of the active histone mark H3K9-Ac were found at the *TFPI-2* promoter regions. In contrast, lower levels of the repressive histone mark H3K9-me3 were found at the same regions (Figure 4C). Together these observations show that the synergistic effect of 5-azaDC and TSA inducing a substantial *TFPI-2* reexpression implies that both DNA methylation and histone deacetylation play important roles in *TFPI-2* silencing in OCSL.

### Effects of *TFPI-2* on the growth of OSCC cells

**Figure 5:**
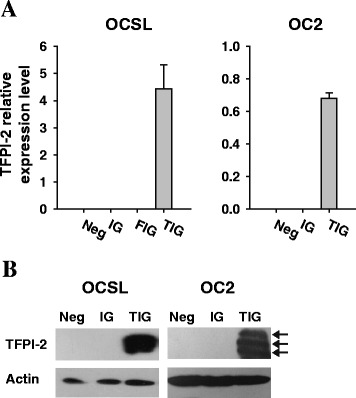
**Reexpression of** ***TFPI-2*** **in OSCC cells by gene transfer. (A)** RT-PCR analysis of TFPI-2 expression in OSCC cells. Experiments were performed in triplicate and the results are shown as the changes in the expression relative to IOSE. Neg, untransduced cells; IG, cells transduced with a control lentiviral vector; FIG, cells transduced with a firefly luciferase-expressing lentiviral vector; TIG, cells transduced with a TFPI-2-expressing lentiviral vector. Error bars indicate standard deviations. **(B)** Western blot analysis of ectopic TFPI-2 overexpression in OSCC cells. ECM proteins were extracted from the cells for Western blot analysis and TFPI-2 isoforms with molecular weights of 27, 31, and 33 kDa due to different extents of glycosylation are indicated with arrows. Beta-actin was used as a loading control.

**Figure 6:**
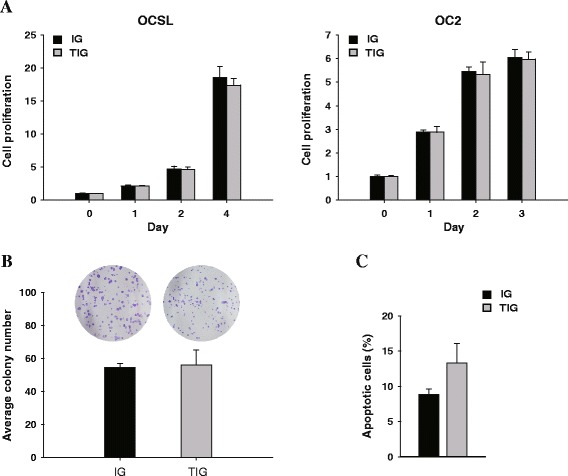
**Effects of TFPI-2 on the growth of OSCC cells. (A)** Effect of TFPI-2 on the cell proliferation of OSCC cells was determined by MTS assay. **(B)** Quantitative analysis of the number of colonies formed using the colony formation assay. OC2 cells transduced with TIG exhibited no change in the number of colonies formed. **(C)** Effect of TFPI-2 on apoptosis of OC2 cells was determined by cell cycle analysis. Error bars indicate standard deviations.

The observation of *TFPI-2* silencing in OSCC tissues and cells inspired us to examine the tumor-suppressive role of TFPI-2 in OSCC. We prepared OSCC cells transduced with *TFPI-2*-expressing lentiviral vector (TIG) and confirmed the reexpression of *TFPI-2* in the cells by qRT-PCR (Figure 5A). Western blot analysis also revealed the presence of TFPI-2 triplets in the ECM of TIG-transduced OSCC cells, while no such protein expression was observed in IG-transduced or untransduced cells (Figure 5B). The effect of restoring *TFPI-2* expression in OSCC was examined by performing cell proliferation, colony formation, and apoptosis assays. Restoration of *TFPI-2* in OSCC cells did not significantly affect the cell proliferation rate, the number of colonies being formed, or the percentage of apoptotic cells (Figure 6).

### TFPI-2 suppresses OSCC cell invasion and blocks MMP-2 activity to reduce tumor metastasis *in vivo*

**Figure 7:**
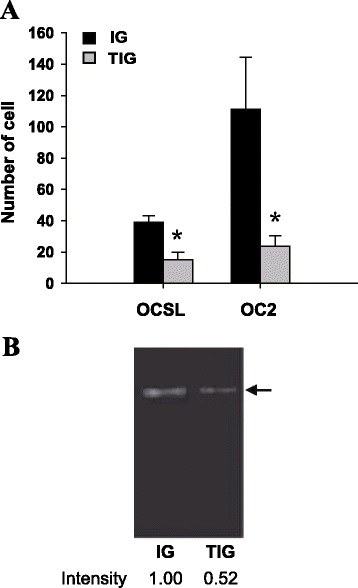
**Inhibitory effect of TFPI-2 on OSCC invasion and MMP-2 enzymatic activity. (A)** *In vitro* cell-invasion assay. Cells that penetrated the Matrigel-coated filter were fixed, stained, and counted in at least three fields of view under a microscope. Comparison between IG and TIG groups was evaluated by Student’s *t*-test, and *P* < 0.05 (*) was considered statistically significant. Error bars indicate standard deviations. **(B)** The enzymatic activity of MMP-2 was determined by collagen zymography. Conditioned medium containing secreted collagenases was collected from the transduced OC2 cell cultures and subjected to collagen zymography. The position of MMP-2 (62 kDa) is indicated by an arrow.

A Matrigel invasion assay was performed to determine whether TFPI-2 has an inhibitory effect on OSCC cell invasion. It was found that TFPI-2 significantly abolished the invasiveness of OCSL and OC2 cells (Figure 7A). Interestingly, the migratory ability did not differ significantly between IG- and TIG-transduced OSCC cells (data not shown), highlighting the ability of TFPI-2 to inhibit ECM degradation during the process of metastasis. Collagen zymography was performed on conditioned medium from the transduced OC2 cells in order to examine whether tumor-secreted proteases were targeted by TFPI-2 inhibition. A less-dense band was found at the position corresponding to the protein size of active MMP-2, demonstrating that the enzymatic activity of MMP-2 was reduced in TIG-transduced cells (Figure 7B).

**Figure 8:**
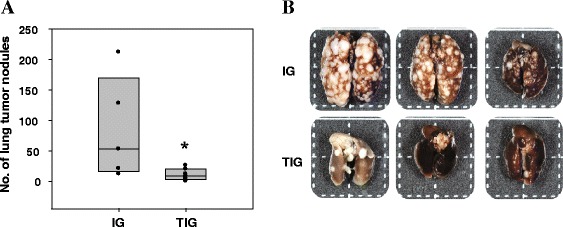
**TFPI-2 reduced OSCC metastasis** ***in vivo.*** **(A)** IG- or TIG-transduced OC2 cells were intravenously injected into the tail veins of athymic BALB/c nude mice (*n* = 5 in the IG group; *n* = 7 in the TIG group). Forty days after tumor cell inoculation, the number of pulmonary metastatic nodules derived from circulating cells in each mouse was counted. The Mann–Whitney U test was used to compare the numbers of tumor nodules between groups. *, *P* = 0.023. **(B)** Representative photograph of lung tissues excised from the mice.

It could be reasoned that TFPI-2 restoration counteracted tumor invasion by negatively regulating MMP-2 activation. To confirm the role of TFPI-2 in the suppression of OSCC metastasis, we performed an experimental metastasis assay by intravenously injecting transduced OC2 cells into nude mice. The number of lung metastatic nodules derived from the circulating cells in each mouse was counted 40 days after tumor-cell inoculation. As shown in Figure 8, far fewer metastatic nodules developed in the TIG group compared to the IG group, demonstrating that TFPI-2 overexpression restrained OC2 metastasis and alleviated tumor malignancy.

## Discussion

In this study we applied a high-throughput methylation array to investigate the DNA methylation status in oral tissue specimens. Comparison of gene methylation profiling between normal oral and OSCC tissues has suggested that *TFPI-2* is hypermethylated in OSCC tissues, an observation that has been confirmed by qMSP and pyrosequencing assays. In agreement with the concept that *TFPI-2* methylation is an early event in esophageal carcinogenesis [40], a significant difference in methylation level of *TFPI-2* was found between normal oral tissues and tumor tissues at early stages, further validating the clinical value of *TFPI-2* methylation profiling for the early detection of OSCC. In addition, the *TFPI-2* methylation level in tumor tissues was higher at the P4 stage than at earlier stages (P1 and P2). Most of the 11 CpG sites analyzed by pyrosequencing assay displayed significant differences in methylation level between early and late stages, indicating the trend of increased methylation with progressive oral tumorigenesis.

While it is clear that *TFPI-2* methylation profiling allows clinical samples to be discriminated, the difference in *TFPI-2* mRNA expression between tumor and normal tissues was not as convincing as was expected (data not shown), mostly due to the heterogeneity of OSCC tissue specimens, in which both *TFPI-*2-expressing and nonexpressing cells were present [41]. Therefore, we measured *TFPI-2* mRNA expression in OSCC cell lines and determined whether *TFPI-2* down-regulation was caused by DNA methylation and histone deacetylation. We successfully reactivated *TFPI-2* expression in OCSL cells but not in OC2 cells after combined treatment with 5-azaDC and TSA. In addition, the extent of *TFPI-2* restoration in OCSL was comparable to that in TIG-transduced OCSL (3.5-fold vs 4.4-fold changes in *TFPI-2* expression relative to IOSE). It is thus possible that for cells that are nonresponsive to 5-azaDC and TSA, such as OC2, the alternatives of viral or non-viral vector-mediated gene transfer for achieving sufficient *TFPI-2* restoration are candidates for OSCC therapy.

The mechanism underlying the effect of DNA methylation on the repression of *TFPI-2* in breast cancer cell lines has been reported previously [42]. Sequence analysis of the *TFPI-2* promoter region identified a potential Kruppel-like factor 6 (KLF6) binding site, suggesting that the aberrant DNA methylation in the KLF6 binding site diminishes the binding of transcription factor KLF6 to the *TFPI-2* promoter to decrease *TFPI-2* expression. Furthermore, Konduri *et al.* reported that methyl-CpG-binding protein 2 (MeCP2) was associated with the methylated *TFPI-2* promoter using a ChIP assay in human glioma cells [32]. The loss of MeCP2 from the activated *TFPI-2* after combined treatment with 5-azaDC and TSA delineated the interplay between histone deacetylation and DNA methylation in the gene-silencing machinery. Together our findings corroborate the earlier conclusion that both promoter methylation and chromatin histone modification play important roles in *TFPI-2* down-regulation.

Consistent with our *in vitro* data showing that TFPI-2 restoration neither suppressed cell proliferation nor promoted cell apoptosis, the tumor size did not differ significantly between IG- and TIG- transduced OSCC cells in a subcutaneous OSCC xenograft model in animals (data not shown). Although TFPI-2 has been found to reduce the cell proliferation rate in various human tumors [40,43–46] by triggering apoptosis [24,44], it has been suggested by some that TFPI-2 has no antiproliferation effect [47–49]. Therefore, the effect of TFPI-2 on cell proliferation and apoptosis may be complex and cell-type specific.

In the present study we demonstrated that TFPI-2 restoration could significantly abolish the invasiveness of OCSL and OC2 cells, but had no inhibitory effect on cell migration. Although TFPI-2 was previously shown to reduce tumor cell invasiveness, the findings regarding its influences on cell migration have been controversial [40,43,45,47,48]. As an inhibitor of ECM degradation, TFPI-2 has been described as an indirect inhibitor of MMPs via plasmin inhibition [27,45,47]. Our data also showed that TFPI-2 counteracts tumor invasion by negatively regulating MMP-2 activation, consistent with the finding that TFPI-2 is associated mainly with the inhibition of ECM degradation [13]. As also reported by Izumi *et al.* [27], TFPI-2 suppressed cell invasion via the down-regulation of the level of active MMP-2 to interfere with the process of tumor metastasis. In addition, we successfully established a metastatic tumor model in animals by the intravenous injection of *TFPI-2*-expressing OC2 cells, and observed a marked inhibitory effect of TFPI-2 in the formation of lung tumor nodules, demonstrating that *TFPI-2* is a silenced tumor suppressor gene in OSCC.

## Conclusions

The significance of the reported differential pattern of *TFPI-2* hypermethylation between normal oral and OSCC tissues makes it a promising biomarker for the early detection of OSCC. *TFPI-2* expression in OSCC can be reactivated after combined treatment with 5-azaDC and TSA, revealing a synergistic regulation of DNA methylation and histone deacetylation in *TFPI-2* silencing. Our data suggest that *TFPI-2* is a down-regulated tumor suppressor gene in OSCC, and that loss of *TFPI-2* expression is a key event in oral tumorigenesis.
